# Selective excitation and imaging of ultraslow phonon polaritons in thin hexagonal boron nitride crystals

**DOI:** 10.1038/s41377-018-0039-4

**Published:** 2018-06-27

**Authors:** Antonio Ambrosio, Michele Tamagnone, Kundan Chaudhary, Luis A. Jauregui, Philip Kim, William L. Wilson, Federico Capasso

**Affiliations:** 1 Center for Nanoscale Systems, Harvard University, Cambridge, MA 02138 USA; 2 Department of Physics, Harvard University, Cambridge, MA 02138 USA; 3 Harvard John A. Paulson School of Engineering and Applied Sciences, Harvard University, Cambridge, MA 02138 USA

## Abstract

We selectively excite and study two new types of phonon-polariton guided modes that are found in hexagonal boron nitride thin flakes on a gold substrate. Such modes show substantially improved confinement and a group velocity that is hundreds of times slower than the speed of light, thereby providing a new way to create slow light in the mid-infrared range with a simple structure that does not require nano-patterning. One mode is the fundamental mode in the first Restrahlen band of hexagonal boron nitride thin crystals on a gold substrate; the other mode is equivalent to the second mode of the second Restrahlen band of hexagonal boron nitride flakes that are suspended in vacuum.

The new modes also couple efficiently with incident light at the hexagonal boron nitride edges, as we demonstrate experimentally using photo-induced force microscopy and scanning near-field optical microscopy. The high confinement of these modes allows for Purcell factors that are on the order of tens of thousands directly above boron nitride and a wide band, with new perspectives for enhanced light-matter interaction. Our findings demonstrate a new approach to engineering the dispersion of polaritons in 2D materials to improve confinement and light-matter interaction, thereby paving the way for new applications in mid-infrared nano-optics.

## 2D materials: Ultraslow phonon polaritons

Applying flakes of hexagonal boron nitride (h-BN) to a gold-coated substrate provides a platform that can slow light in the mid-infrared range, according to US researchers. Antonio Ambrosio and coworkers from Harvard University in the USA transferred a 121 nm-thick flake of the 2D material h-BN to a silicon substrate coated with a 100 nm layer of gold. They discovered that the h-BN-gold material system supports two types of phonon polariton guided modes, one of which has a very large group refractive index in the mid-infrared (191 at a wavenumber of 1500 cm^−1^) indicating slow light propagation. Scanning Near-field Optical Microscopy (s-SNOM) and Photo-Induced Force Microscopy (PiFM) were used to characterize the modes. PiFM allowed unprecedented high-resolution imaging of these modes in the 795 and 1900 cm^−1^ spectral range.

## Introduction

Hexagonal boron nitride (h-BN) is an out-of-plane anisotropic (but in-plane isotropic) material with two very strong phonon-polariton bands where the permittivity becomes negative. In the first Restrahlen band (RS1, 780–830 cm^−1^), the relative out-of-plane permittivity *ε*_||_ is negative, while in the second restrahlen band (RS2, 1370–1610 cm^−1^), the relative in-plane permittivity $\varepsilon _ \bot$ is negative. Due to these optical properties, thin h-BN flakes support guided modes, which have been observed experimentally via both far-field and near-field methods^1–14^.

The guided modes in the RS2 band have been largely investigated by means of scattering-type near-field optical microscopy (s-SNOM). The situation is more complicated in the RS1 band due to the lack of continuous-wave (CW) laser sources that can be used to implement the necessary interferometric detection schemes of s-SNOM. In this work, we describe how to selectively excite the more confined modes in the RS1 and RS2 bands. We also demonstrate the possibility of full hyperspectral nano-imaging of modes in the RS1 band by means of photo-induced force microscopy^15–18^ (PiFM). Moreover, a direct comparison of (PiFM) and s-SNOM is obtained by imaging the modes of the RS2 band with both techniques implemented on the same platform.

**Fig. 1:**
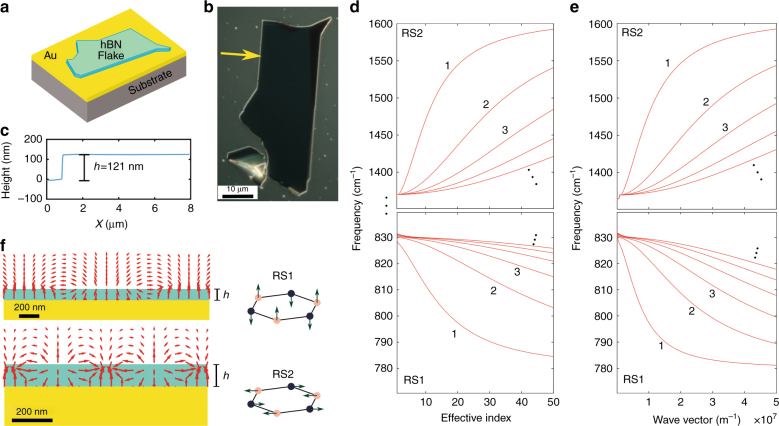
Slowing down polaritonic modes by mirroring. **a** Schematic diagram of our sample. A silicon substrate is coated with a 100-nm-thick e-beam evaporated gold coating. A 121-nm-thick h-BN flake is deposited on the gold film. **b** Optical image of the h-BN crystal flake, which was obtained using a phase-contrast microscope. The yellow arrow points to the edge that is imaged in this work. **c** AFM profile of the flake at the edge. **d** Polaritonic mode effective index. **e** Polaritonic mode dispersion. **f** Field configuration for the first mode in each of the RS bands. The main effect of the gold substrate (a perfect conductor at these wavelengths) is to reduce the polariton group and phase velocities by inhibiting less-confined modes. For example, in the RS2 band at 1500 cm^−1^, the effective refractive index of the first guided mode in our configuration is *n*_eff_ = 12.9 while the effective group index is *n*_*g*_ = 191. The same mode on a SiO_2_ substrate would have *n*_eff_ = 4.02 and *n*_*g*_ = 101

Our sample consists of a 121-nm-thick h-BN flake that was exfoliated from a bulk h-BN crystal using adhesive tape and transferred onto a gold-coated substrate (silicon +500 nm wet thermal oxide +100 nm e-beam evaporated gold with 5 nm chromium for adhesion; Fig. 1a–c).

Both the RS1 and RS2 bands are in wavelength ranges where the gold substrate shows very high-negative permittivity values (on the order of thousands). Therefore, the gold film can be accurately approximated as a perfect electric conductor (PEC). Experiments on h-BN on gold substrates were also recently performed in the RS2 band and reported in ref. ^19^.

The dispersion in this system (which relates the wavevector *k* and the angular frequency *ω*) is given by (Supplementary Information):

1 $$\frac{{\varepsilon _d\sqrt {\frac{{\varepsilon _ \bot }}{{\varepsilon _\parallel }}k^2 - \varepsilon _ \bot \frac{{\omega ^2}}{{c^2}}} }}{{\varepsilon _ \bot \sqrt {k^2 - \varepsilon _d\frac{{\omega ^2}}{{c^2}}} }}\tanh \left( {h\sqrt {\frac{{\varepsilon _ \bot }}{{\varepsilon _\parallel }}k^2 - \varepsilon _ \bot \frac{{\omega ^2}}{{c^2}}} } \right) + 1 = 0$$

where *ε*_*d*_ is the relative dielectric permittivity of the superstrate (here, it is air, for which *ε*_*d*_ = 1), *c* is the speed of light and *h* is the h-BN flake thickness. By inspection of the equations, in the RS bands where ε_||_ and $\varepsilon _ \bot$ have opposite signs, infinite solutions can be found for arbitrarily large values of *k*. Such solutions (polaritonic modes) are called hyperbolic since they originate from the opposite sign of the transverse and longitudinal components of the permittivity tensor. Figure 1e graphically represents the first solutions of the dispersion equation, while Fig. 1d shows the same for the modal effective index *n*_eff_ = *kcω*^−1^.

There are two noticeable effects that are related to placing the flake on gold instead of on a dielectric substrate: (1) Equation (1) is exact, while the case of a dielectric substrate is solved in the literature by assuming large wavenumbers only^4^; (2) Due to the mirror symmetry, the possible guided modes for the flake on gold correspond to the odd modes of a flake that is twice as thick and suspended in air (Supplementary Information). This last point is important since most of the time, the guided mode that is reported and discussed in the literature is the first even guided mode in the RS2 band, which is much less confined than the first odd mode in the same band. As a practical example, at 1500 cm^−1^, the effective refractive index of the first guided mode in our configuration is $n_{{\rm eff}} = \frac{\omega }{k} = 12.9$ while the effective group index is $n_g = \frac{{\partial \omega }}{{\partial k}} = 191$. The same flake suspended in vacuum would have *n*_eff_ = 3.96 and *n*_*g*_ = 100, and that on a SiO_2_ substrate would have *n*_eff_ = 4.02 and *n*_*g*_ = 101. The mode at 1500 cm^−1^ on gold has a phase velocity that is three times slower than the equivalent mode that is excited on a dielectric substrate, with a calculated propagation length of 1750 nm. The high values of the achieved phase and group effective indices are both interesting in nanophotonics. A high phase index allows the realization of miniature resonators and subwavelength optics, while a high group index is associated with electric field enhancement, which is useful for sensing. In addition, the combination of slow light and high-mode confinement substantially enhances the Purcell factor for an emitter that is placed above the h-BN. Numerical calculations show that for an emitter that is placed 30 nm above h-BN and oriented along *z*, the Purcell factor exceeds 15,000 for the RS2 band and 70,000 for the RS1 band.

Due to the lack of continuous-wave mid-IR lasers in the RS1 band, nano-imaging with s-SNOM at a single wavelength in the RS1 band has been impossible so far. The only near-field imaging that has been reported in the h-BN RS1 band has been hyperspectral imaging using nano-FTIR with broadband sources^3,7^. However, this kind of measurements are limited in spectral (maximum travel range of the interferometer) and spatial resolution (number of pixels in the image). In this work, using PiFM in the RS1 band, we show both nano-imaging at a single wavelength and hyperspectral imaging with 512 × 512 pixels and a spectral resolution of 2 cm^−1^.

## Results

**Fig. 2:**
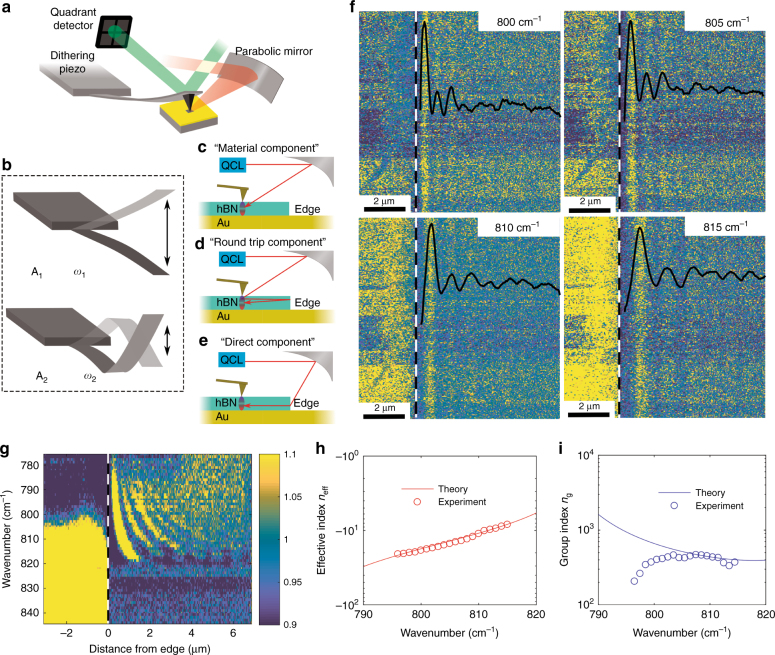
PIFM hyperspectral nano-imaging in the RS1 band. **a** Schematic diagram of the PiFM setup. A mid-IR laser is focused on the AFM tip close to the sample surface by means of a parabolic mirror. The mechanical oscillations of the cantilever are detected by means of a quadrant photo-detector. **b** First and second mechanical eigenmodes of an AFM cantilever. The second eigenmode has a resonance frequency of ~6.3 times the resonance frequency of the first eigenmode. **c**–**e** The contributions of the three possible paths to the measured signal. The material component (**c**) is due to the local polarizability of the material. The roundtrip component **d** is due to a polaritonic guided mode that originates from the AFM tip and couples back to the tip after being reflected from the flake edge. The direct component (**e**) originates from a polaritonic mode that is directly coupled into the flake via scattering at the edge. **f** PiFM imaging (and profile) of the fringes that are induced by the interference of the guided modes with the material component at the tip location for various frequencies (wavenumbers) of the illuminating light in the first RS band. The white dashed line represents the position of the flake edge. **g** Plot of the PiFM signal intensity as a function of the distance from the edge and the illuminating light frequency. This plot is obtained by averaging each of the images at a specific illuminating wavelength along the vertical axis (parallel to the flake edge) and is used to retrieve the experimental data that are plotted in **h** and **i**. Since our imaging is hyperspectral, all the images at a specific wavelength are obtained in a single scan where a full spectrum is recorded at each position (pixel) of the scanned area. **h**, **i** Analytical (Equation 1) and measured modal phase and group effective index as functions of the illuminating light frequency. The group velocity is ~500 times slower than the speed of light in free space. In Fig. 2i, the deviation of the experimental data from the theoretical prediction is a consequence of reduced laser power below 800 cm^−1^. This results in the deterioration of the signal-to-noise ratio in the imaging and a large uncertainty in the calculation of the derivative $\frac{{\partial \omega }}{{\partial k}}$, which provides the group refractive index

Our photo-induced force microscopy setup for imaging in the RS1 band (based on a commercially available microscope from Molecular Vista; Fig. 2a) and its operation have been recently described in ref. ^18^. In brief, the feedback on the second eigenmode of an atomic force microscope (AFM) cantilever, oscillating at *ω*_2_ (Fig. 2b), is used to stabilize the tip-sample distance during scanning and extract the sample morphology. The first eigenmode of the cantilever is excited by the interaction forces between the sample and the tip, which are induced by the external laser source. This interaction is detected in the cantilever mechanics when the laser intensity is modulated at the frequency difference between the first and second eigenmode resonances^20,21^. In this case, the laser (QCL from Block Engineering) is p-polarized, the pulse width is 20 ns, and the light wavenumber (wavelength) can be tuned between 795 and 1900 cm^−1^. The probe is a gold-coated tip with a resonance frequency of ~300 kHz. The second eigenmode is used for tip-sample distance stabilization with a tapping modulation amplitude of a few nanometers and a typical setpoint between 70 and 80%.

PiFM is one of the recent approaches that show the potential of mechanical detection for optical nano-imaging^18,22–24^. With our imaging procedure, we can fix the laser wavelength and scan (nano-imaging) or we can swipe the laser wavelength between 795 and 1900 cm^−1^ at each pixel of the image (hyperspectral imaging) with a minimum wavelength step of 2 cm^−1^. Recording a hyperspectral image enables us to retrieve the phonon-polariton dispersion in the RS1 band.

Figure 2f–g show the results of the experimental detection of the first mode of the RS1 band via PiFM. The origin of the light-driven interaction (force) that is probed in PiFM is usually described in terms of an interaction force between a light-induced dipole in an AFM tip and its mirror image in the sample. This interaction has been discussed to be representative of the dispersive part of the material polarizability (*material* component; Fig. 2c)^25,26^. The material component mostly depends on the optical properties of the topmost material and is the only component that contributes to the detected signal when no guided modes or localized resonances are supported by the sample. However, when phonon polaritons are present, a local spatial modulation of the electric field at the surface is also present^27^. This also affects the tip-sample interaction and may produce, by means of interference with the material component, characteristic features (fringes) in the optical image (polariton components). Importantly, we are using the term components to refer to the interaction between the AFM tip and a local field that has a complex amplitude (amplitude and phase) that is associated with the specific phonon path. In our configuration, two polariton components are possible in principle: (1) a *roundtrip* component (Fig. 2d), which produces fringes in the optical image with periodicity of half the phonon-polariton wavelength. This component originates from the AFM tip, which launches a polariton by scattering the illuminating beam. This polariton propagates as a circular wave to the edge of the flake, where it is reflected back to the AFM tip; (2) a *direct* component (Fig. 2e), which has the same periodicity as the polariton mode and originates from scattering at the sharp edges of the h-BN flakes. A more detailed description of each component is provided below.

Figure 2f shows examples of PiFM imaging close to the edge of the flake at various incident light frequencies. Here, the experimental periodicities correspond to the direct component only, and their values are those that are expected for the first mode in the RS1 band. In this case, each of the images in Fig. 2f comes from a single-hyperspectral dataset. Supplementary movie 1 shows how the spacing of the polaritonic fringes changes as a function of the illuminating light frequency in the RS1 band: longer wavelengths correspond to a larger fringe periodicity. Figure 2g shows the fringe periodicity as a function of the illuminating light wavelength. The data that are reported in Fig. 2g also enable us to obtain the guided wavevector, which is subsequently used to calculate the effective phase and group indices. Figure 2h, i shows the theoretical and experimental values of the effective phase and group refractive index of the first mode in the RS1 band. The experimental results of the phase velocity are in excellent agreement with the theory. However, there is disagreement between the computed and measured group velocities. This effect is larger at shorter wavenumbers. The origin of this disagreement is that calculating the group index involves computing a derivative, which is very sensitive to the uncertainty in the measurement of the phase velocity. The uncertainty in deriving the phase velocity from the experimental data results from having a lower signal and thus fewer fringes at the lower edge of the RS1 band, which is where the disagreement is observed. The phase and group indices have very high values and opposite signs (negative group velocity). For instance, at 800 cm^−1^, *n*_eff_ = 17.58 and *n*_*g*_ = −729. The mode is strongly confined (calculated propagation length of 600 nm), with a group velocity that is ~500 times lower than the speed of light in free space. These values are completely consistent with previous spectral analysis of such modes^3^.

For the imaging and analysis of the modes in the second RS band (1370–1610 cm^−1^), we used both s-SNOM and PiFM. For this study, a commercially available s-SNOM microscope was used (Neaspec). PiFM measurements were made by utilizing home-implemented detection electronics to extract the cantilever dynamics on the same optical setup. Then, s-SNOM and PiFM images in the RS2 band were obtained nearly simultaneously (one after the other) on the same microscope with the same AFM probe (a Pt-Ir coated cantilever, with 70 kHz resonance frequency) and the same laser, namely, a QCL laser (DayLight). The laser is operated in continuous wave (CW) emission for s-SNOM and intensity modulated (50/50 duty cycle) for the PiFM imaging. This approach enables direct comparison between PiFM and s-SNOM imaging results of the polaritonic modes. The tapping modulation amplitude of the first eigenmode, once the tip is in contact, ranges between 70 and 90 nm, with a typical setpoint of 80%. When operating in PiFM, the second eigenmode of the AFM cantilever is used for PiFM detection.

**Fig. 3:**
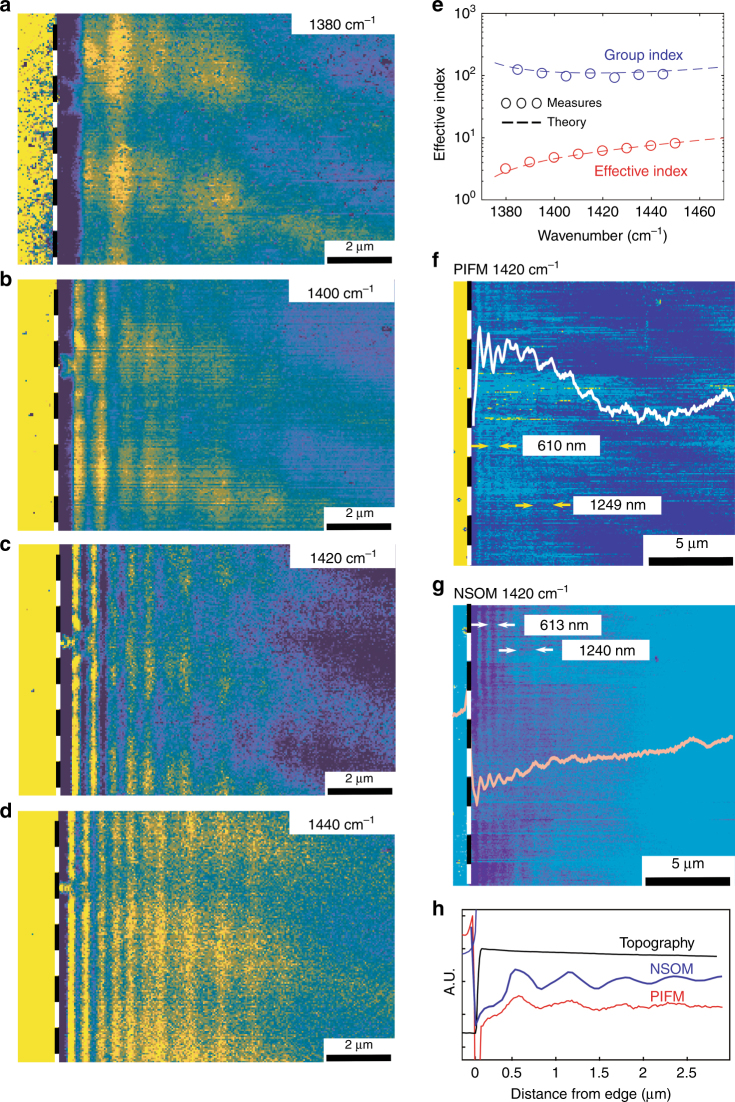
s-SNOM and PiFM images in the RS2 band. **a**–**d** s-SNOM images (amplitude from the second harmonic) of the h-BN polaritonic modes in the second RS band for various illuminating light frequencies. The long periodicity along the fast scan axis results from a residual interference of the illuminating laser light scattered components. **e** Group and phase indices that are calculated from the s-SNOM data. Each image at an illuminating wavelength is averaged parallel to the flake edge (represented by the white dashed line). Then, the periodicity is used to retrieve the guided-mode wavevector. **f**–**h** Comparison between s-SNOM and PIFM images in the second RS band. Both images show fringes with single (1240 nm) and double periodicity (610 nm), which correspond, respectively, to the direct and roundtrip components of the first polaritonic guided mode. In the PiFM image and the s-SNOM amplitude image, the fringes have maxima and minima in the same positions, thereby proving the similarity between the origins of the two signals

Figure 3a–d shows four s-SNOM scanning results at various wavelengths in the RS2 band (amplitude maps from the pseudoheterodyne configuration that was extracted from the second harmonic of the AFM cantilever oscillation). These images are used to calculate the group and phase indices in the RS2 band (Fig. 3e). From these images, we observe both the half periodicity of the roundtrip component and the single periodicity of the direct component, which becomes the only visible component far from the flake’s edge. PiFM and s-SNOM (amplitude map) show peaks in the same positions for both the roundtrip component and the direct component (Fig. 3f–h). This result agrees with the theoretical work that was reported in literature in which both the PiFM signal and the s-SNOM signal (amplitude) have been predicted to describe the real part of the local polarizability (dispersive behavior)^28–33^. In line with this description, the polaritonic mode represents only an extra modulation of the local field at the sample surface (or, alternatively, a local spatially modulated effective polarizability).

However, the s-SNOM setup, combined with the CW QCL and the pseudoheterodyne detection, also enables the retrieval of the local field phase, rather than just the amplitude. Mapping both the amplitude and phase is crucial for obtaining a more comprehensive picture of the polaritonic components (see the discussion below).

## Discussion

The existence of both direct and roundtrip components has been discussed for flat h-BN^3,9^ and experimentally observed in the RS2 band^34,35^. Our imaging procedures enable us to observe the interference fringes that result from both of these components, thereby allowing for the first time nano-imaging of the direct component that is launched by the h-BN flake edge in the RS1 band. The absence of a roundtrip component while imaging the mode in the RS1 band can be explained as follows: the electric field distribution of the polaritonic modes in the RS1 band is more “internal” than in the RS2 band, with large field components deeper inside the material (Fig. 1f)^7^. This condition reduces the coupling efficiency (in both injection and detection) for the modes that are launched by the external tip. In addition, the RS1 mode has a vertical electric field distribution across the entire sample thickness (Fig. 1f). This also couples well with an incident wave at the edge of the crystal (direct component). In contrast, the RS2 mode has a more complex field distribution, which reduces the edge coupling efficiency and makes the roundtrip and direct components similar in terms of excitation and detection efficiency.

The material component is the signal that results from the local polarizability of the material. It can be represented by a complex number $\tilde A_M$ whose strength depends on the local material response under the AFM tip, and the phase depends on the relative position of the AFM tip with respect to the external illumination. The AFM tip is fixed with respect to the illuminating light. Therefore, the material component is expected to have the same complex value everywhere on a material, e.g., on the h-BN flake. In a complex plane representation, the complex values of the material component should distribute themselves around a single value, unless the scanned material has defects on the surface.

The direct component is due to the phonon-polariton wave that is launched by the flake edge. It propagates from the edge to the tip, where it is scattered. This component can be represented by a complex number $\tilde A_D = A_D \cdot e^{ - ik_{{\rm PP}}x}$, where *k*_pp_ is the phonon-polariton wavenumber. The direct component spatially modulates the field in the medium, which interferes with the material component under the tip to create intensity maxima and minima (fringes) with periodicity *λ*_pp_ (phonon-polariton wavelength). The amplitude of the direct component under the AFM tip is set by the internal coupling efficiencies and propagation losses, while the phase depends on the distance *x* between the edge and the AFM tip. For any other interference between a constant reference (in this case, represented by the material component) and a propagating wave, the representation in the complex plane (*Re*, *Im*) corresponds to a spiral with the internal point set by the most delayed configuration (the tip far from the flake edge).

The roundtrip component is not a standing wave, as is often reported in the literature, even though its periodicity (twice the direct component) is the same as that of a standing wave between the tip and the edge. The roundtrip component originates from the phonon-polariton that is launched by the AFM tip, which returns to the AFM tip after propagation and reflection at the flake edge. The complex amplitude of the modulation is related to such component changes by $\widetilde {A_S} = A_{{\rm RT}}x^{ - \frac{1}{2}}e^{ - i2k_{{\rm PP}}x}$ since as the AFM tip moves a distance *L* toward the edge during scanning, the optical path in a roundtrip changes by 2 *L*. This results in the half-periodicity that is observed for this component. Analogously to the direct component, the observed fringes originate from the interference of the roundtrip component and the material component under the tip. The representation of the interference in the complex plane is again a spiral that converges to a point.

**Fig. 4:**
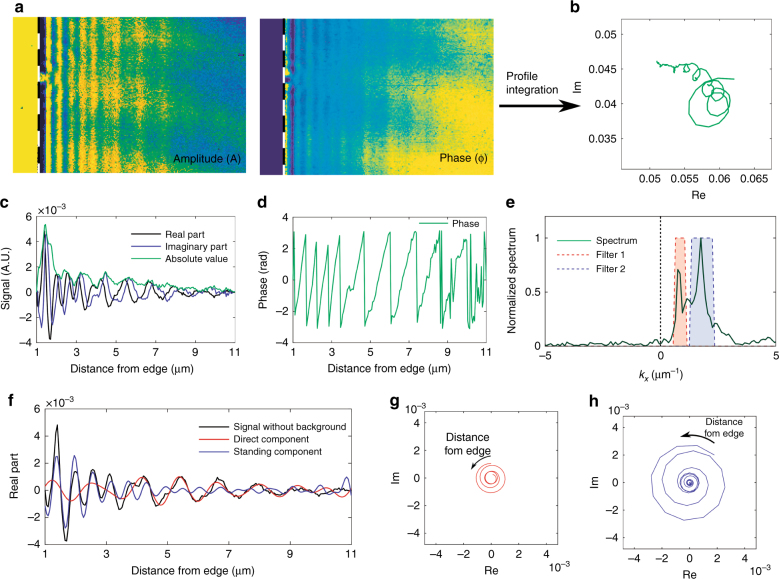
Extraction of the three polaritonic components with pseudoheterodyne s-SNOM. **a** Amplitude (*A*) and phase (*φ*) of the s-SNOM second harmonic signal close to the flake edge. These values can be combined and represented in a complex plane (*Re, Im*) = (*A* cos*φ, A* sin*φ*). **b** In this representation, the material component produces a distribution of close-together values; the direct and the roundtrip components instead contribute with values that are distributed along the arms of two spirals. From this distribution of values, each single component can be retrieved as described below. **c**, **d** Spatial average (along *y*) profiles for amplitude, phase, the real and imaginary parts of the polaritonic wave are obtained from the maps in **a**. **e** Complex Fourier transform that was obtained from the profiles that were reported in **c** and **d**. This spectrum shows two peaks, which correspond to the single and double periodicities of the direct and roundtrip components. Peaks appear only in the positive-wavenumber region, which directly corroborates the description of both the direct and roundtrip components as propagating waves that propagate in the same direction (away from the edge). **f** Inverse Fourier transform of **e** after applying two bandpass filters to restrict the spectrum region (dashed areas of the spectrum in **e**). **g**, **h** show the phasor evolution (spiral) of the electric field that results from the direct and roundtrip components, respectively

The s-SNOM setup is based on pseudoheterodyne detection. This enables the simultaneous imaging of both the local field amplitude and phase, which makes it possible to retrieve the individual components, as shown in Fig. 4.

Figure 4a shows the amplitude (*A*) and phase (*φ*) of the s-SNOM second harmonic signal close to the flake edge. These values can be combined and represented in a complex plane (*Re, Im*) = (*A* cos*φ, A* sin*φ*) (Fig. 4b). In this representation, the material component produces a distribution of close-together values, as described above. The direct and roundtrip components instead contribute with values that are distributed along the arms of two spirals. From this distribution of values, each component can be retrieved as described below.

The spatially averaged (along *y*) profiles for amplitude, phase, and the real and imaginary parts of the polaritonic wave are obtained from the maps in Fig. 4a. These plots are shown in Fig. 4c, d. At a distance from the flake edge, the single periodicity of the direct component is the only one that is visible. This is expected since the direct component is attenuated less in propagation than the roundtrip component that results from a wave that has propagated twice as much for each tip position. The profiles of Fig. 4c, d can be used to calculate the complex Fourier transform, which is shown in Fig. 4e. This spectrum shows two peaks, which correspond to the single and double periodicities of the direct and roundtrip components. Peaks appear only in the positive-wavenumber region, thereby directly corroborating the description of both the direct and roundtrip components as propagating waves that propagate in the same direction (away from the edge).

Once the peaks have been identified, two bandpass filters can be applied to restrict the spectrum region (dashed areas of the spectrum) around the peaks and retrieve the profiles of the direct and roundtrip components via the inverse Fourier transform (Fig. 4f). The obtained signals are complex-valued and depend on the distance of the tip from the edge. We can represent these quantities with a parametric plot (where the distance is the parameter) on the complex plane, which is equivalent to plotting the evolution of the phasor with distance. Figure 4g, h shows these plots for the direct and roundtrip components, respectively. A clear spiral trend is observed, which is due to the complex exponentials $e^{ - ik_{{\rm PP}}x}$ and $e^{ - 2ik_{{\rm PP}}x}$, as discussed above.

We experimentally retrieved the mode dispersion in the first RS band by means of hyperspectral imaging of the modes with PiFM. We experimentally demonstrated that only highly confined modes in the second RS band are excited due to the mirror symmetry that is introduced by the gold substrate. We also measured the mode dispersion in the second RS band by both s-SNOM and PiFM, which were implemented on the same setup. This constitutes an unprecedented comparison between s-SNOM and PiFM, with results that are in line with previous theoretical work on the nature of the signals that are provided by these two techniques.

These results represent an important advance in the engineering of polaritonic modes in vdW materials to achieve strong light-matter interaction in the mid-IR region and a fundamental milestone in high-resolution optical imaging.

## Materials and methods

For photo-induced force microscopy (PiFM) operation, the first eigenmode of the cantilever is excited by the interaction forces between the sample and the tip, which are induced by the external laser source. This interaction is detected in the cantilever mechanics when the laser intensity is modulated at the frequency difference between the first and second eigenmode resonances. In this case, the laser (QCL from Block Engineering) is p-polarized, the pulse width is 20 ns, and the light wavenumber (wavelength) can be tuned between 795 and 1900 cm^−1^. The probe is a gold-coated tip with a resonance frequency of ~300 kHz. The second eigenmode is used for tip-sample distance stabilization, with a tapping modulation amplitude of a few nanometers and a typical setpoint of between 70 and 80%. When a CW QCL laser (DayLight) is used, the laser is intensity modulated (50/50 duty cycle). The tapping modulation amplitude of the first eigenmode, once the tip is in contact, ranges between 70 and 90 nm, with a typical setpoint of 80%. The second eigenmode of the AFM cantilever is used for PiFM detection.

## Electronic supplementary material

Supplementary Information

Supplementary Information Video RS1
