# Supplementary material for: Selective excitation and imaging of ultraslow phonon polaritons in thin hexagonal boron nitride crystals
Source: Light Sci Appl. 2018 Jun 27;7:27. doi: 10.1038/s41377-018-0039-4 (PMC6107022; doi:10.1038/s41377-018-0039-4)
Supplement: Supplementary file 1 — Supplementary Information [file 41377_2018_39_MOESM1_ESM.pdf]

Supplementary Information: Selective excitation and imaging ultraslow phonon polaritons in thin hexagonal boron nitride crystals.

Antonio Ambrosio,[1,2,‡,*] Michele Tamagnone,[3,‡] Kundan Chaudhary,[3] Luis A. Jauregui,[2] Philip Kim,[2] William L. Wilson,[1] Federico Capasso.[3,*]

[1]Center for Nanoscale Systems, Harvard University, Cambridge, Massachusetts 02138, USA

[2]Department of Physics, Harvard University, Cambridge, Massachusetts 02138, USA

[3]Harvard John A. Paulson School of Engineering and Applied Sciences, Harvard University, Cambridge, Massachusetts 02138, USA

[‡] These Authors contributed equally to this work

*Corresponding Author, email: ambrosio@seas.harvard.edu, capasso@seas.harvard.edu

h-BN modelling

h-BN is modelled as an anisotropic material with the following relative permittivity tensor:

$$\bar{\bar{\varepsilon}}_r = \begin{pmatrix} \varepsilon_x & 0 & 0 \\ 0 & \varepsilon_y & 0 \\ 0 & 0 & \varepsilon_z \end{pmatrix}, \quad \varepsilon_x = \varepsilon_y$$

$$\varepsilon_x = \varepsilon_y = \varepsilon_\perp = \varepsilon_{\infty\perp}\left(1 - \frac{\left(\omega_{LO,\perp}\right)^2 - \left(\omega_{TO,\perp}\right)^2}{\omega^2 - j\omega\Gamma_\perp - \left(\omega_{TO,\perp}\right)^2}\right)$$

$$\varepsilon_z = \varepsilon_\parallel = \varepsilon_{\infty\parallel}\left(1 - \frac{\left(\omega_{LO,\parallel}\right)^2 - \left(\omega_{TO,\parallel}\right)^2}{\omega^2 - j\omega\Gamma_\parallel - \left(\omega_{TO,\parallel}\right)^2}\right)$$

$$\omega_{TO,\perp} = 1370\ cm^{-1} \equiv 7.299\ \mu m \qquad \text{(S1)}$$

$$\omega_{LO,\perp} = 1610\ cm^{-1} \equiv 6.211\ \mu m$$

$$\varepsilon_{\infty\perp} = 4.87 \qquad \Gamma_\perp = 5\ cm^{-1}$$

$$\omega_{TO,\parallel} = 780\ cm^{-1} \equiv 12.82\ \mu m$$

$$\omega_{LO,\parallel} = 830\ cm^{-1} \equiv 12.048\ \mu m$$

$$\varepsilon_{\infty\parallel} = 2.95 \qquad \Gamma_\parallel = 4\ cm^{-1}$$

These expressions can be cast into a Lorentz model with the following parameters:

$$\varepsilon_x = \varepsilon_y = \varepsilon_\perp = \varepsilon_{\infty\perp} + \frac{\varepsilon_{Lorentz\perp}(\omega_{0\perp})^2}{(\omega_{0\perp})^2 + j\omega 2\delta_\perp - \omega^2}$$

$$\varepsilon_z = \varepsilon_\parallel = \varepsilon_{\infty\parallel} + \frac{\varepsilon_{Lorentz\parallel}(\omega_{0\parallel})^2}{(\omega_{0\parallel})^2 + j\omega 2\delta_\parallel - \omega^2}$$

$$\omega_{0,\perp} = 1370\ cm^{-1} \equiv 7.299\ \mu m$$

$$\varepsilon_{\infty\perp} = 4.87 \qquad \varepsilon_{Lorentz\perp} = 1.8557 \tag{S2}$$

$$\delta_\perp = 2.5\ cm^{-1}$$

$$\omega_{0,\parallel} = 780\ cm^{-1} \equiv 12.82\ \mu m$$

$$\varepsilon_{\infty\parallel} = 2.95 \qquad \varepsilon_{Lorentz\parallel} = 0.3903$$

$$\delta_\parallel = 2\ cm^{-1}$$

Guided modes in a suspended h-BN slab

This section derives the field geometry and effective indexes of the modes in a h-BN slab with thickness $2h$ embedded in a dielectric with permittivity ε_d (in our case air has $\varepsilon_d = 1$, but the symbol will be preserved for completeness). We assume without loss of generality that the modes are propagating in the in-plane x direction, with a guided wavenumber $k_x = n_{eff} k_0$ to be determined.

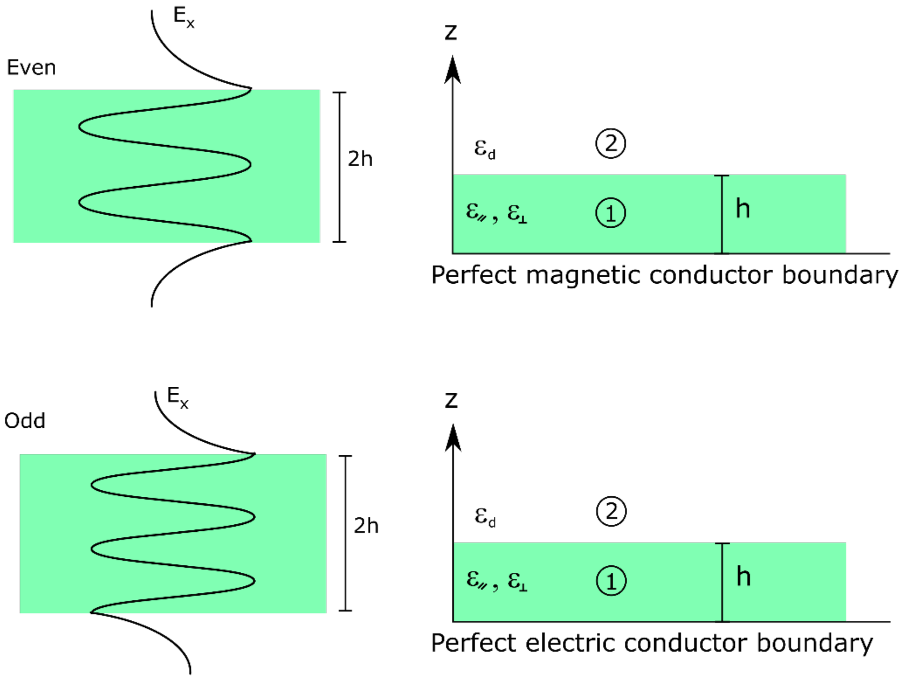

Supplementary Figure 1 | Analytic derivation of modes dispersion. Using the image theorem even and odd modes in an h-BN slab are equivalent to those found in a slab with half the thickness and terminated by perfect magnetic and perfect electric conductor boundary condition respectively.

Supplementary figure 1 shows the geometry of the problem. Using the image theorem, the even modes in a suspended h-BN slab are equivalent to those found in a slab with half thickness placed on a perfect magnetic conductor boundary condition. Similarly, odd modes are analogous to those in a half thickness slab on perfect electric conductor.

We now focus on the perfect electric conductor case, which is the one corresponding to the experiment. Assuming that k_x is the guided wavenumber, then solving Maxwell's equations one can find two counterpropagating plane waves solutions in region 1 (h-BN) and one evanescent wave in region 2 (air). The magnetic and electric fields in region 1 are then given by:

$$\boldsymbol{H_1} = \left(0, H_{y1+}, 0\right)e^{\,ik_x x+\alpha_{z1}z} + \left(0, H_{y1-}, 0\right)e^{\,ik_x x-\alpha_{z1}z}$$

$$\boldsymbol{E_1} = \frac{iH_{y1+}}{\omega\epsilon_0}\left(\frac{\alpha_{z1}}{\varepsilon_\perp}, 0, -\frac{ik_x}{\varepsilon_\parallel}\right)e^{\,ik_x x+\alpha_{z1}z} + \frac{iH_{y1-}}{\omega\epsilon_0}\left(-\frac{\alpha_{z1}}{\varepsilon_\perp}, 0, -\frac{ik_x}{\varepsilon_\parallel}\right)e^{\,ik_x x-\alpha_{z1}z} \qquad \text{(S3)}$$

with:

$$\alpha_{z1} = k_0\sqrt{\frac{\varepsilon_\perp}{\varepsilon_\parallel}n_{eff}^2 - \varepsilon_\perp}\ ,\ \ \alpha_{z2} = k_0\sqrt{n_{eff}^2 - \varepsilon_d}\ ,\ \ k_0 = \frac{\omega}{c} \qquad \text{(S4)}$$

The evanescent wave in region 2 is described as:

$$\boldsymbol{H_2} = \left(0, H_{y2}, 0\right)e^{\,ik_x x+\alpha_{z2}z} \qquad \text{(S5)}$$

$$\boldsymbol{E_2} = \frac{iH_{y2}}{\omega\epsilon_0}\left(-\frac{\alpha_{z2}}{\varepsilon_d}, 0, -\frac{ik_x}{\varepsilon_d}\right) e^{\,ik_x x + \alpha_{z2} z}$$

Due to the presence of the perfect electric conductor, $H_{y1+} = H_{y1-}$. Therefore, the fields in region 1 can be written as:

$$\boldsymbol{H_1} = 2\left(0, H_{y1}\cosh\alpha_{z1}z\,, 0\right)e^{\,ik_x x}$$

$$\boldsymbol{E_1} = \frac{2iH_{y1}}{\omega\epsilon_0}\left(\frac{\alpha_{z1}}{\varepsilon_\perp}\sinh\alpha_{z1}z\,, 0, -\frac{\alpha_x}{\varepsilon_\parallel}\cosh\alpha_{z1}z\right)e^{\,ik_x x} \quad \text{(S6)}$$

Applying the continuity of transversal magnetic and electric field at the interface between region 1 and 2 we find

$$2H_{y1}\cosh\alpha_{z1}d = H_{y2}e^{\,\alpha_{z2}d}$$

$$2H_{y1}\frac{\alpha_{z1}}{\varepsilon_\perp}\sinh\alpha_{z1}d = -H_{y2}\frac{\alpha_{z2}}{\varepsilon_d}e^{\,\alpha_{z2}d} \quad \text{(S7)}$$

Dividing member by member:

$$\frac{\alpha_{z1}\varepsilon_d}{\alpha_2\varepsilon_\perp}\tanh\alpha_{z1}d + 1 = 0 \tag{S8}$$

which is the dispersion relations used in this work. With a similar process, one can find that in the perfect magnetic case (corresponding to even modes in the free-standing h-BN equivalent) the dispersion is:

$$\frac{\alpha_{z1}\varepsilon_d}{\alpha_2\varepsilon_\perp}\coth\alpha_{z1}d + 1 = 0 \tag{S9}$$

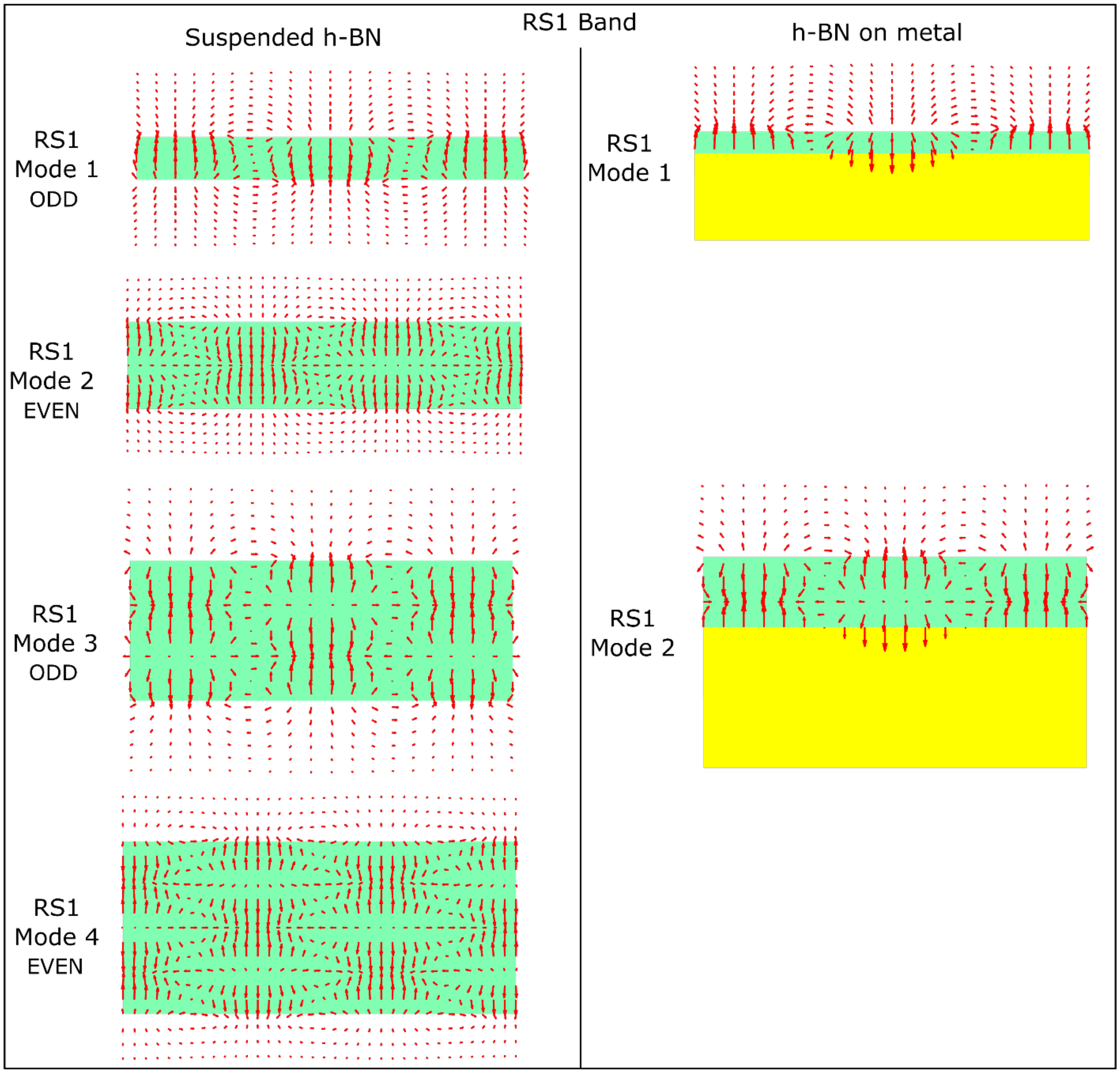

Supplementary Figure 2 | Analogy between modes in suspended h-BN and h-BN on metal in the RS1 band. The first four calculated modes for a suspended h-BN flake are shown. All the modes propagate from left to right. Fields are shown at the frequency of 818 cm^{-1}. The thickness of the suspended flake is $2h = 242\ nm$, the flake on gold has thickness $h = 121\ nm$

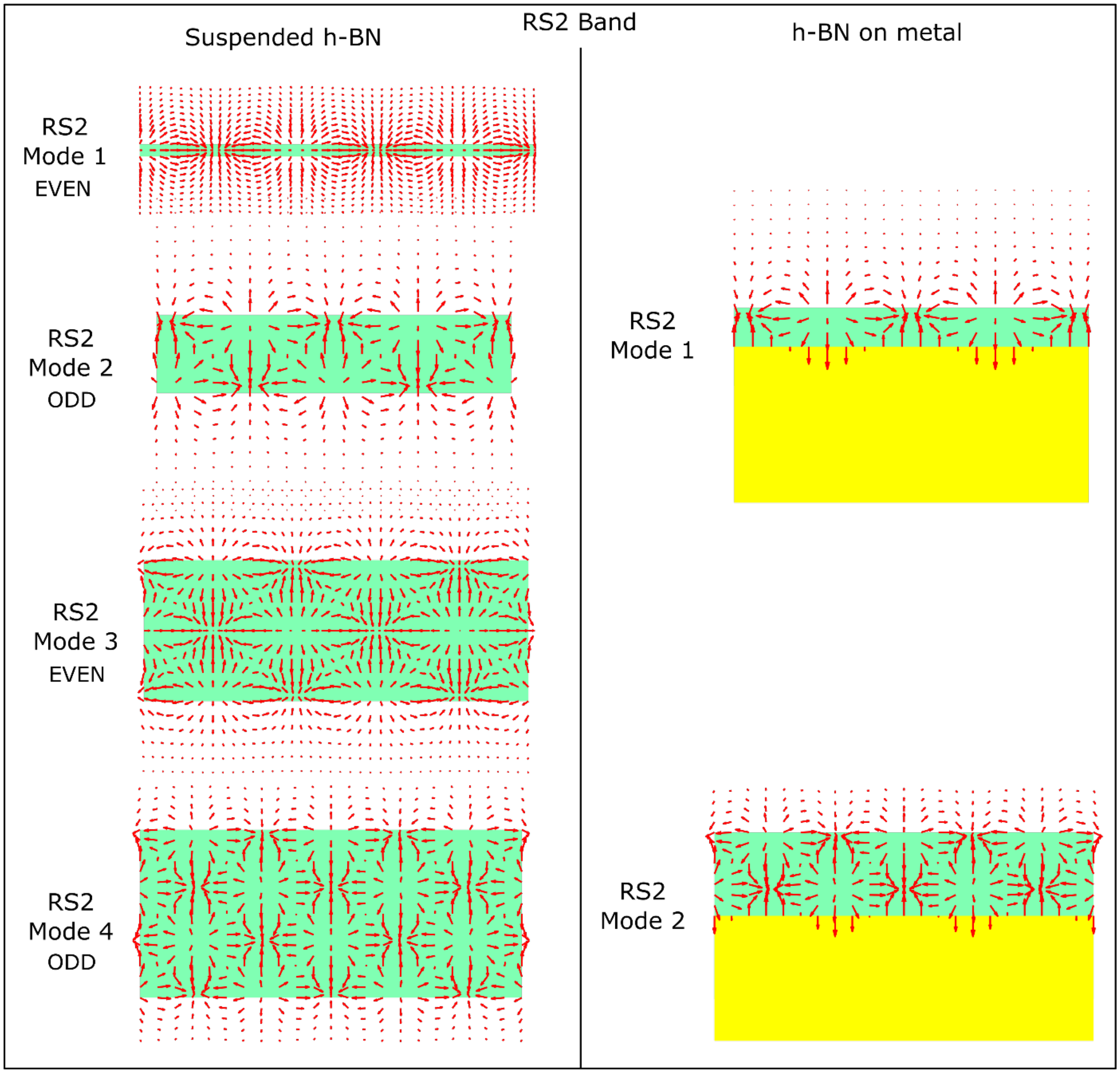

Supplementary Figure 3 | Analogy between modes in suspended h-BN and h-BN on metal in the RS2 band. The first four calculated modes for a suspended h-BN flake are shown. All the modes propagate from left to right. Fields are shown at the frequency of 1500 cm^{-1}. The thickness of the suspended flake is $2h = 242\ nm$, the flake on gold has thickness $h = 121\ nm$

Additional measurements

This section introduces some additional measurements showing the presence of the interference fringes in the s-SNOM amplitude images done in the RS2 band with different harmonics and tip materials (Supplementary Figure 4). For metallic and silicon tips and for the second, third and fourth harmonics both the *direct* and *roundtrip* interference patterns are observed. A defect is visible on the edge, and the circular waves launched by it are also visible.

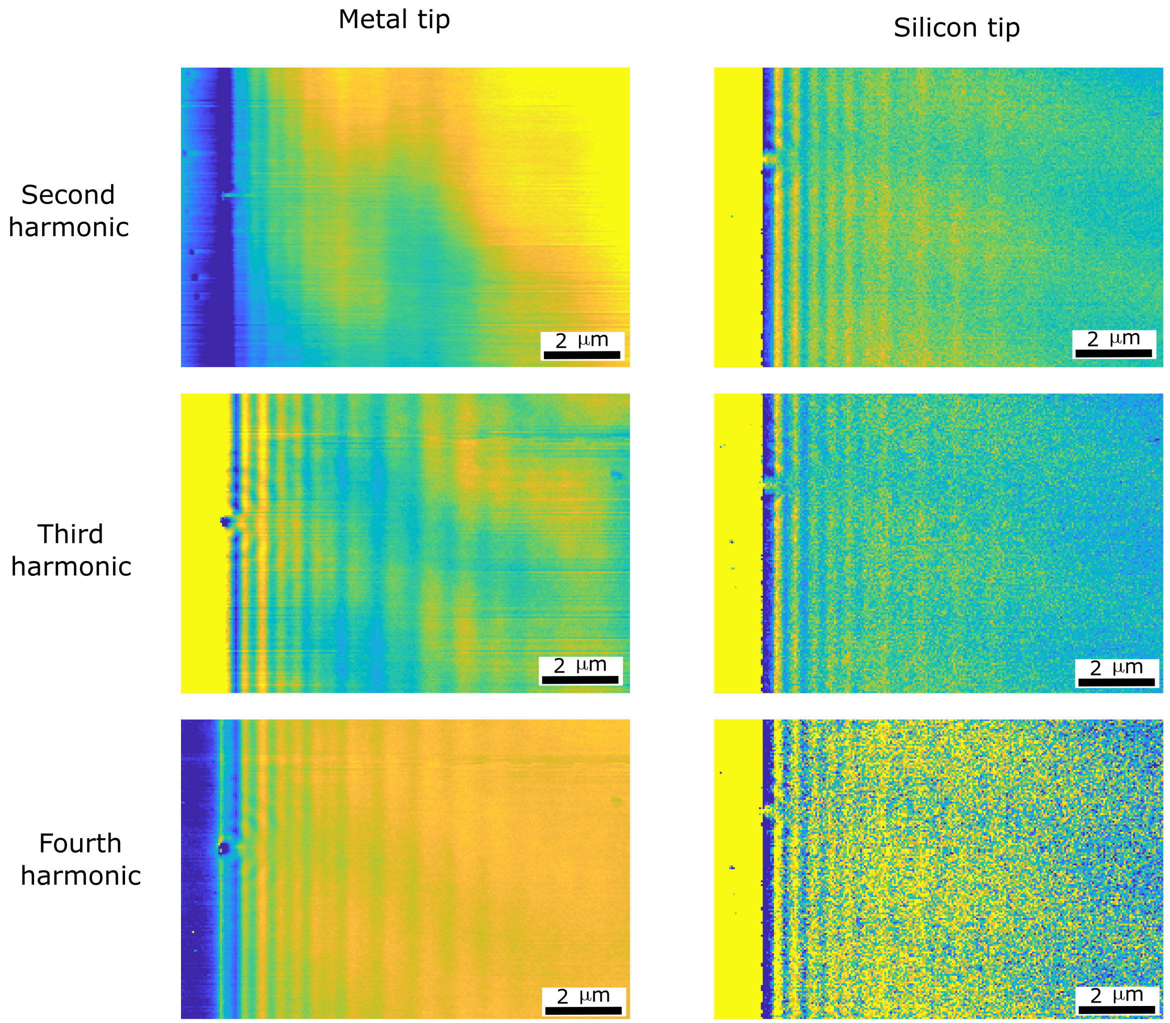

Supplementary Figure 4 | s-SNOM images for different tip materials and harmonics. All the plots represent the s-SNOM amplitude, measured at 1440 cm^{-1}.

Effect of the beam inclination on the direct component

The fringes of the *direct* component observed in the RS1 an RS2 bands are not exactly spaced as the guided wavelength of the mode. This is because the edge moves as the sample is scanned and because the illuminating beam is tilted; there is then a de-phasing of the launched mode. This dephasing can be corrected knowing the direction of the illuminating beam ($\theta = 60°$ with respect to the normal, ϕ =45° with respect to the edge). Since the transversal wavevector ($k_x = \sin(\theta)\cos(\phi)\, k_0$) of the impinging wave then adds to the guided mode to form the fringes, then the correction consists in subtracting $\sin(\theta)\cos(\phi)$ from the measured effective index. However, such effect is small given the large effective indexes.

Computation of Purcell factors

The computation of Purcell factors has been performed using the commercial software Lumerical FDTD. The stack described above is modeled in the software and a dipole source above h-BN and directed along z is used. After the simulation, the Purcell factor is directly extracted from the source results. Supplementary Figure 5 shows the dependence of the Purcell factor as a function of distance from the h-BN layer and wavelength.

Additional PiFM images in the RS1 band

Supplementary Figure 6 shows additional PiFM images in the RS1 band of the sample in a corner region, showing the first real imaging in the RS1 band.

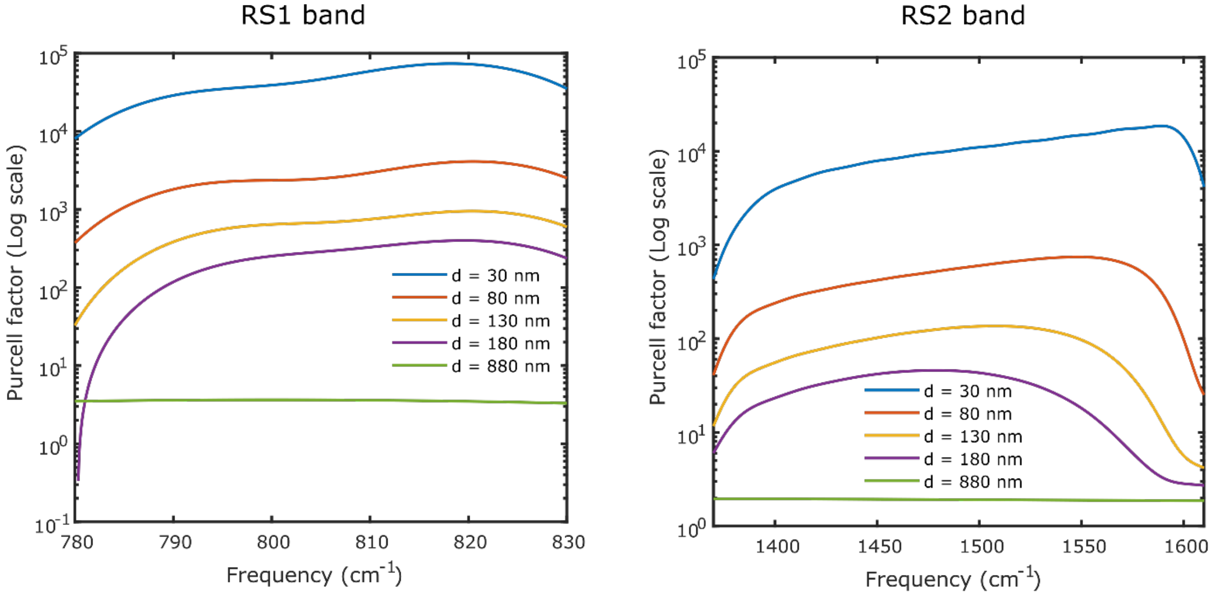

Supplementary Figure 5 | Purcell factor as a function of frequency, for different distances *d* from h-BN. All data is calculated for a z-directed electrical dipole.

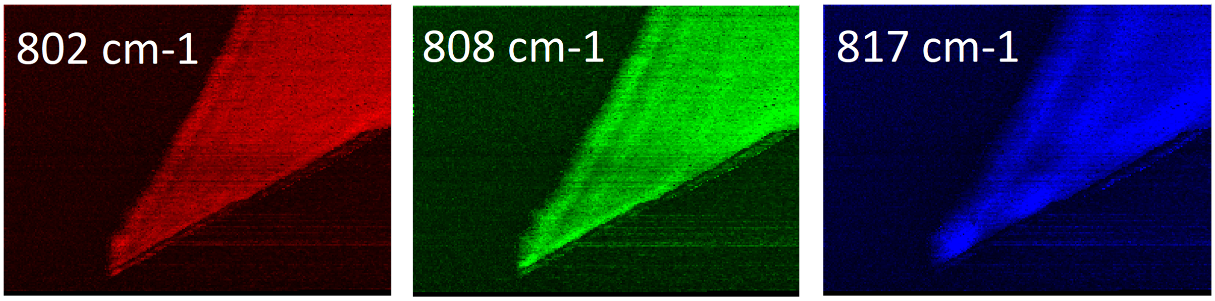

Supplementary Figure 6 | Additional PiFM images in the RS1 band.
